# Autism Spectrum Disorder in Children: Early Signs and Therapeutic Interventions

**DOI:** 10.3390/children11111311

**Published:** 2024-10-29

**Authors:** Turki Abualait, Maryam Alabbad, Imdad Kaleem, Hadia Imran, Hamid Khan, Mubin Mustafa Kiyani, Shahid Bashir

**Affiliations:** 1 College of Applied Medical Sciences, Imam Abdulrahman Bin Faisal University, Dammam 34212, Saudi Arabia; msalabbad@moh.gov.sa; 2 Department of Medical Rehabilitation and Long-Term Care, Al-Ahsa Health Cluster, Al-Ahsa 31982, Saudi Arabia; 3 Department of Biosciences, COMSATS University Islamabad (CUI), Islamabad 45550, Pakistanhadiaqureshi@yahoo.com (H.I.); 4 Department of Biological Sciences, Faculty of Basic and Applied Sciences, International Islamic University, Islamabad 44000, Pakistan; hamid.phdbt99@iiu.edu.pk; 5 Shifa College of Medical Technology, Shifa Tameer-e-Millat University, Islamabad 44000, Pakistan; mubin.scmt@stmu.edu.pk; 6 Neuroscience Center, King Fahad Specialist Hospital, Dammam 32253, Saudi Arabia; shahidb@moh.gov.sa

**Keywords:** autism spectrum disorder, diagnosis, screening, signs, intervention

## Abstract

Autism Spectrum Disorder (ASD) is a complex neurodevelopmental condition characterized by challenges in communication, social interaction difficulties, and repetitive behaviors that can hinder a child’s development. The growing prevalence of autism necessitates early detection and effective intervention strategies. This review summarizes the current knowledge of early indicators of ASD, including brain development markers and behavioral signs visible in infants. It investigates diagnostic processes, emphasizing the importance of timely detection at 18 to 24 months using established screening tools. We discuss a variety of therapeutic approaches, including behavioral interventions, educational strategies such as music therapy, and technological advancements such as speech-generating devices. Furthermore, we investigate pharmacological options for treating associated symptoms, emphasizing the lack of targeted medications for core ASD symptoms. Finally, we present evidence highlighting the positive effects of early intervention on developmental outcomes, advocating for individualized treatment plans to enhance the well-being of children with ASD. This comprehensive overview aims to inform ongoing ASD research and clinical practices.

## 1. Introduction

Autism Spectrum Disorder (ASD) is a heterogenous neurodevelopmental condition manifested by difficulties in communication, social interaction, and engagement, accompanied by stereotypic, repetitive behaviors or focused interests that impacts various areas of a child’s development [1]. Estimates of how common ASD is have risen over the past few decades, highlighting the significance of early identification and the need for effective intervention strategies [1]. It is still uncertain how much of the rise in ASD prevalence reflects a true increase in cases. Enhanced identification and diagnosis, along with the expansion of diagnostic criteria across various editions of the Diagnostic and Statistical Manual of Mental Disorders, are probable factors influencing prevalence estimates [1,2]. Studies suggest that some children can be accurately diagnosed with ASD as early as age two [3], although more subtle cases might not become apparent until later. Despite increased awareness of early signs, the typical age of diagnosis remains between 4 and 5 years [4]. The etiology of ASD remains elusive, but recent findings suggest a complex interplay of genetic, epigenetic, and environmental causes [5]. Key risk factors include being male and having a family history of ASD, with the likelihood of recurrence in younger siblings ranging from 7% to 19% [6,7], compared to 1.5% in the general population [2]. The level of risk depends on the closeness of familial relationships. For example, a recent study from Sweden reported a tenfold increase in relative risk if a full sibling has ASD, compared to a twofold increase if the condition is present in a cousin [8]. Environmental mechanisms potentially linked to ASD include inflammation, oxidative stress, and hormonal disruption [9,10].

Evidence increasingly supports that early diagnosis and targeted interventions can positively impact various developmental domains for children with ASD [11]. Research indicates that early detection not only enhances developmental outcomes but also improves social integration and overall long-term well-being [6]. Despite the importance of early detection of ASD, variations in clinical procedures and the lack of standardized diagnostic tools lead to discrepancies in the early recognition, identification and treatment of ASD [12]. The inconsistency in the efficacy of ASD therapeutic interventions proven by certain studies that show variable success rates and distinct outcomes based on factors like comorbid conditions and symptom severity [13,14]. These inconsistent findings underscore the importance of future studies aimed at enhancing diagnostic criteria and intervention procedures, ensuring that treatments are timely and customized.

In this review, we present a summary of ASD early predictors, neurodevelopmental characteristics, and interventions. It aims to clarify the complexity of early diagnosis, the neurobiological variations identified in newborns, and the impact of various therapy techniques. By combining existing research findings and emphasizing gaps in clinical practice, this review intends to inform and guide future studies and treatments, eventually improving early identification and treatment outcomes for children diagnosed with ASD.

## 2. Search Strategy, Identification of the Studies, and Study Characteristics

This narrative review provides an overview of research concerning ASD in children, specifically focusing on early signs and therapeutic interventions. To identify relevant studies, four databases were utilized: Google Scholar, PubMed, Web of Science, and Cochrane Library. The following Boolean search syntax was applied: ((Autism Spectrum Disorder) OR (ASD)) OR (autism) AND ((((((“early signs”)) OR (“behavioral indicators”)) OR (“therapeutic interventions”)) OR (“intervention”)) OR (“early diagnosis”)) OR (screening). Subsequently, various filters were applied, including: text availability (full text), species (humans), languages (English), and a publication period of the last thirty years. Finally, additional selection was conducted using specific inclusion and exclusion criteria, such as:

(i) Publications in English;
(ii) Studies published between 1990 and 2024;
(iii) Focus on early predictors and therapeutic interventions for ASD;
(iv) Study design: randomized controlled trials or systematic reviews.

Studies were excluded if they did not meet these criteria or if they lacked a direct focus on early signs or therapeutic interventions pertinent to ASD.

## 3. Brain Development and Early Indicators of ASD: Evidence from Longitudinal Studies

Numerous prospective longitudinal research investigating potential brain-based biomarkers state revealed various variations in neural development that appear around or prior to the emergence of early behavioral indicators of ASD [12]. Multiple techniques have been utilized to assess the structure and function of the brain in infants, each offering a distinct perspective regarding neural development. Insights from MRI studies, alongside early findings on syndrome-associated autism using animal models, point to variations in the growth and development of neural progenitors and neurogenesis [13]. Changes in the balance between inhibitory and excitatory processes at neuronal and synaptic activity may also affect the initial formation of functional brain circuitry in ASD, as indicated by a variety of electroencephalogram (EEG) alterations observed during the initial year of life [14]. Whilst behavioral characteristics develop, the ways in which infants interact with their environment likely further influence the growth of experience-dependent neural circuitry.

### 3.1. Magnetic Resonance Imaging Findings

Prospective magnetic resonance imaging (MRI) studies have revealed significant differences in the early neural development of infants who are later diagnosed with ASD. Notably, excessive brain growth, characterized by enlarged cerebral cortex volume, occurs within first two years of life among infant siblings who will be identified with ASD [15]. These infants show rapid expansion of the cortical surface area, particularly in the occipital, temporal, and frontal lobes, from six to twelve months, which heads further excessive brain development from twelve months to two years [15]. Additionally, accelerated amygdala growth is observed in infants with siblings diagnosed with ASD at six and twelve months, compared to those with fragile X syndrome and neurotypical development [16].

A study of fifty infants exhibited growth in subcortical regions among four to six month old infant siblings, irrespective of diagnosis [17]. Another longitudinal study revealed that by twelve months, infants who would later be diagnosed with ASD had larger subcortical structures compared to those with early language delays or without autism [18]. Furthermore, research comparing 270 infant siblings to 108 low-likelihood infants showed that an enlarged corpus callosum at six months predicted future ASD-related behaviors, although variations in the thickness of the corpus callosum among diagnosed and non-diagnosed children decreased by the time they reach two years old, indicating a dynamic developmental mechanism [19].

In research involving 92 infant siblings, fractional anisotropy measurements indicated differences in white matter development between those who were later diagnosed with ASD and those who were not. Infants with ASD exhibited higher fractional anisotropy at 6 months, followed by a more gradual change up to twenty-four months [20]. Similarly, a study of 116 infant siblings revealed that those later diagnosed with ASD displayed variations in white matter network efficiency at 6 months [21]. Differences in white matter maturation in specific brain regions have been linked to particular autism-related behaviors [13].

A longitudinal study involving 221 high-risk infant siblings and 122 low-risk infants revealed that elevated extra-axial cerebrospinal fluid (CSF) levels at 6 months predicted later ASD, with raised levels persisting until twenty-four months [22]. CSF is thought to contain growth factors that support neuronal proliferation and may help clear metabolites that can impact brain function.

Resting-state functional MRI (fMRI) studies during sleep identified disturbances in thalamocortical connectivity and language-related networks as early as 1.5 months in infant siblings, regardless of future diagnosis [23]. At 6 months, atypical functional connectivity patterns in fMRI were linked to an increased likelihood of autism identification by age two [24].

While most MRI studies focus on group differences, a few have examined individual-level predictions. An algorithm at 6 months combining extra-axial CSF, brain volume, age, and sex were predictors of autism diagnosis with 66% sensitivity and 68% specificity [22]. Cross-validated machine learning analyses of resting-state fMRI at the same age achieved 82% sensitivity and 100% specificity for predicting autism [24]. Additionally, assessments of cortical surface area from six to twelve months predicted outcomes with 88% sensitivity and 95% specificity [15].

### 3.2. Electrophysiological Predictors

Auditory brainstem response recordings, spontaneous EEG, and event-related potentials (ERPs) have demonstrated differences in timing, amplitude, and spectral power in infants which could act as predictors for ASD. An examination of prior auditory brainstem responses from an auditory screening involving 139,154 neonates (321 later diagnosed with ASD) revealed prolonged variations in the phase of auditory brainstem responses and the latency of wave V-negative have been observed in those diagnosed with ASD [25]. Machine learning studies on spontaneous EEG data from 99 high-risk infant siblings and 89 low-risk infants indicated that EEG complexity and various power spectral densities could predict later autism [26]. Although one study linked heightened alpha-band EEG connectivity at 14 months was linked to later autism diagnosis [27], another found this connectivity related only to limited interests and repetitive behaviors [28]. Among eight-month-old infants, heightened cortical reactivity to repeated tones was associated with later autism diagnosis, evidenced by the reduced suppression of 40–60 Hz gamma responses and elevated 10–20 Hz inter-trial coherence [29]. AT six months, infants who were later diagnosed with ASD exhibited weaker ERPs in response to facial expressions and shorter visual attention spans to faces compared to peers without ASD [30]. Additionally, infants aged six to ten months exhibited reduced inter-trial coherence in the theta frequency band while processing visual facial stimuli [31]. Infants diagnosed with autism at 8 months displayed a diminished N290 ERP, which is typically responsive to face stimuli [32]. Predictive accuracy for subsequent ASD enhanced when autism polygenic scores were included in the logistic model assessing predictions derived from N290 latency responses to both face and non-face stimuli [32].

In a small randomized controlled trial (RCT) involving infant siblings aged nine to eleven months (*n* = 33), EEG measurements were utilized to evaluate the impact of an early intervention aimed at enhancing social interaction during caregiver-infant engagements. Infants who were given this caregiver-delivered intervention exhibited developmental patterns in both EEG (frontal theta power) and ERP (P400 response to faces) that aligned more closely with those of neurotypical infants, differing from those who did not receive the intervention [33].

In another study involving 91 infant siblings and 40 low-risk infants aged eight months, shorter durations of EEG microstates associated with social attention were predictive of future autism diagnosis [34]. Additionally, a study with 161 high-risk infant siblings and 71 low-risk infants utilized independent components analysis to identify patterns across various cognitive and adaptive measures, ASD-related behaviors, and ERP responses to eye gaze shifts, aiming to uncover cross-domain patterns linked to subsequent autism diagnoses [35].

Research on individual-level predictions indicates that electrophysiological biomarkers may be effective for early autism screening, demonstrating high sensitivity and specificity as early as within the first three months of life. In a longitudinal study involving siblings and low-risk infants, EEG power trajectories— especially in the delta and gamma frequency bands—from three to twelve months accurately predicted later ASD diagnoses among infant siblings, achieving a sensitivity of 0.82 and specificity of 0.86 [26]. Additionally, an algorithm incorporating non-linear EEG characteristics (such as entropy and detrended fluctuation assessments) was able to predict autism diagnoses in infant siblings at just three months, demonstrating a sensitivity of 0.82 and a specificity of 0.99 [36]. An analysis of clinical auditory brainstem response recordings from zero to three months found that extended wave-V latency could differentiate between thirty infants later diagnosed with ASD and thirty matched control infants, with a sensitivity of 0.70 and specificity of 0.80 [25].

### 3.3. Insights from Near-Infrared Spectroscopy

While near-infrared spectroscopy (NIRS) offers lower spatial resolution compared to fMRI, it is advantageous for use with infants and toddlers during activities [37]. In a NIRS study focusing on five-month-old infants comprising 16 infant siblings and 13 low-risk infants—researchers examined brain activity in response to social videos (such as the behaviors of a female actor) and contrasted these with reactions to non-social stimuli (like images of cars). Low-risk infants exhibited greater activation in the right posterior temporal cortex in contrast to their infant siblings [38]. Another analysis involving infants aged four to six months comprising twenty infant siblings and sixteen low-risk infants revealed that those later diagnosed with ASD exhibited diminished activation in the inferior frontal and posterior temporal areas when viewing social videos, along with decreased activation in response to vocal sounds and increased activation in response to environmental noises within the left temporal regions [39]. An investigation involving thirty-two infants who were six months old (including fourteen infant siblings) revealed that those later diagnosed with ASD had diminished responses to speech sounds in bilateral temporal and frontal areas [40]. However, no studies have yet reported individual-level predictions using NIRS.

## 4. Behavioral Signs of ASD in Infants

Infants aged twelve months and younger exhibit various behavioral predictors of autism. These involve variations in attention, prelinguistic communication development, emotional expression, temperament, social interaction, sensory sensitivity and habituation, motor skills, play with toys, and the presence of restricted and repetitive behaviors [12].

### 4.1. Attention Differences

Attention differences are a hallmark of autism, affecting an infant’s capacity to focus on environmental elements. These differences vary by context and complexity, such as between orienting and joint attention. Early attention patterns can influence development and promote neural specialization in certain areas. Infants later diagnosed with autism often show diminished attention to social cues [41], like faces and voices [42], which may negatively impact social growth and is affected by genetic factors [43]. Studies indicates that infants diagnosed with autism at six, nine, and twelve months exhibited lower attention to faces when caregivers interacted with them, but this was not observed during singing or play [44]. Unlike neurotypical infants and those without autism, they did not differentiate their gaze between caregivers and strangers [45]. Additionally, a study discovered that toddlers later diagnosed with ASD spent more time looking at individuals during predictable interactions [46].

Reduced responsiveness to their own names is a social attention patterns of autism in toddlers [47,48] and serves as a pre diagnostic indicator, with predictive value starting at 9 months and increasing through infancy [49], although it may not specifically indicate autism until twenty-four months [50]. Joint attention, where a caregiver and child focus on the same object, is crucial for language development [51]. Previous study involving 482 infant siblings, and 178 low-risk infants found that those who later developed ASD had reduced joint attention initiation at twelve months [52]. Nevertheless, a smaller study involving fifty-seven infant siblings did not observe this reduction [53]. Additionally, an alternative study indicated that fourteen-month-old siblings who were later diagnosed with ASD initiated joint attention less frequently and had decreased coordination of these behaviors with vocalizations compared to their non-autistic peers [54].

Infant siblings who are twelve months old and later diagnosed with ASD show a delayed response in shifting their attention from fearful faces [55]. Additionally, a longitudinal study involving 83 infant siblings and 53 low-risk infants found that those later diagnosed with ASD demonstrated asymmetric and prolonged disengagement from geometric stimuli at twelve months, with prolonged left-directed disengagement linked to increased irritability and challenges in being soothed [56].

### 4.2. Prelinguistic Communication Development

Differences in prelinguistic communication development have been observed between infants later diagnosed with ASD and those who were not. During their first year, infants typically move from non-syllabic to syllabic vocalizations, showing the emergence of canonical syllables around seven months and increasing thereafter. By nine to twelve months, infants who will later be diagnosed with ASD generate fewer canonical vocalizations and more non-canonical ones [57]. Atypical vocalization patterns, particularly reduced canonical babbling, have been noted in these infants and also in those with later language delays [58]. Caregiver responses are more likely to occur in reaction to canonical vocalizations, which are essential for refining babbling and enhancing communication [59]. Consequently, early vocal production differences may lead to diminished social feedback, impacting communication and language development. Infant siblings later diagnosed with ASD often show fewer socially directed vocalizations [60]. Unusual crying patterns have also been identified in these infants as early as one month old [61].

Infants who are subsequently diagnosed with ASD show a unique trajectory in gestural development. Between eight and fourteen months, they demonstrate a decreased use of gestures, especially deictic gestures like pointing, and demonstrate less gesture-vocal coordination compared to neurotypical peers, infant siblings without a diagnosis of ASD, and those with language delays [62,63]. Gesture usage at twelve months is indicative of an autism diagnosis and correlates with both expressive language skills at that age and later language abilities [64].

### 4.3. Affective Expressions and Temperament

Variations in emotional expressions and temperament have been noted in infants between six and nine months old who are later diagnosed with ASD, such as heightened negative affect and regulatory difficulties [65,66]. However, study findings can vary due to differing methodologies (e.g., caregiver reports vs. clinical observations) [67]. A longitudinal study involving analyzing 473 infant siblings and 176 low-risk infants revealed that lower caregiver-reported positive affect and attentional shifting were predictors of a later autism diagnosis (*n* = 129), a pattern that remained consistent from six to twenty-four months [68]. By twelve to eight-teen months, toddlers who were later diagnosed with ASD showed reduced levels of positive affect and reduced smiling [67,68]. Additionally, decreased regulatory capacity and heightened negative affect have been observed in infants who are twelve months old and above [66], and in toddlers later diagnosed with attention-deficit/hyperactivity disorder (ADHD) [69,70]. Between eight-teen and thirty-six months, toddlers with ASD display heightened neutral affect and diminished social engagement, positive anticipation, and attentional regulation, particularly in shifting attention, as reported by caregivers and through clinical observations [67,69,71,72]. By six months, infants later diagnosed with autism display persistent alterations in social engagement, for instance less eye contact with caregivers [44]. At nine months, these infants demonstrate reduced eye gaze, facial expressions, gestures, and vocalizations through interactions [62]. By twelve months, they tend not to redirect their focus towards their caregiver’s touch and exhibit lower dyadic synchrony, which is correlated with later language abilities [73,74].

### 4.4. Sensory Responses and Interests

The DSM-5 diagnostic criteria for ASD include variations in sensory responses and preferences under the category of restricted interests and repetitive behaviors [12]. These differences may manifest as hyposensitivity to repeated stimuli or hypersensitivity to new stimuli, along with altered sensory engagement with the environment. Habituation, which refers to the diminishing reaction to repeated sensory input followed by a recovery response to new stimuli, can be affected in these children [12]. Reduced habituation rates can lead to either increased or decreased sensitivity. Research on sensory sensitivity and habituation in young children with ASD has utilized caregiver observations, eye-tracking data, and EEG research.

Based on caregiver reports, changes in sensory processing have been identified in infants as early as six to twelve months, often occurring before a diagnosis of ASD and the emergence of restricted and repetitive behaviors [75,76]. This connection becomes more evident during the second year of life [77,78], and is evident in both social and non-social situations [79]. EEG investigates and eye tracking have assessed the timing and intensity of sensory responses to repeated stimuli in infants, revealing links to later autism diagnoses or autism-related behaviors across visual [80,81], auditory [29], and tactile domains [82]. Additionally, a lack of sensitivity to intersensory coordination suggests unusual sensory integration [83]. Therefore, sensory sensitivities can be identified by eight to ten months and become more predictive of an autism diagnosis between twelve to twenty-four months, increasingly aligning with the diagnostic criterion of hypersensitivity to sensory input [80,81].

### 4.5. Motor Skills Development and Object Exploration

In infants, the development of motor skills provides opportunities for exploring objects and engaging in interactive play, which are essential for cognitive and language growth. By six to nine months, infants later diagnosed with ASD show delays in sitting, pull-to-sit, reaching to grasp, and goal-oriented reaching [84]. Delays in fine and gross motor skills can be seen by six months and are predictors of future language abilities [84,85]. Additionally, difficulties in postural control, such as delayed sitting, can also emerge by six months and persist over time [86].

Alterations in object use have been noted in infants who later receive an autism diagnosis. While their capacity to anticipate the movements of hidden objects remains intact [87], reduced exploratory behaviors are evident in those aged 10 months [88]. By 18–24 months, these infants exhibit less exploratory play and have unusual toy interests, such as fascination with vacuums or specific hats [89].

Repetitive behaviors begin to appear by 9 months, particularly in the form of atypical visual inspection of objects, such as carrying them near to the face [90]. By twelve months, infants diagnosed with ASD show increased frequency of stereotyped motor behaviors, repetitive object manipulation, and head movements [91]. Self-injurious behaviors have also been reported in infant siblings by nine months, though these behaviors are not exclusive to those later diagnosed with ASD [91].

## 5. Early Diagnosis and Screening of ASD

ASD can sometimes be recognized as early as eight-teen months, but a reliable diagnosis is typically achievable by age two through an experienced professional. Since there is currently no medical test available for ASD, clinicians rely on the child’s developmental background and behavior for diagnosis. The National Center on Birth Defects and Developmental Disabilities (NCBDD) advises screenings at 9 months, 18 months, and 24 or 30 months, while the American Association of Pediatrics (AAP) advises including ASD screening in routine checkups at 18 and 24 months [92]. Parents are encouraged to be actively involved in this process. Understanding developmental milestones is essential for diagnosing ASD. The Centers for Disease Control and Prevention (CDC) provides guidelines that outline developmental milestones for children from birth to five years, covering social, emotional, language, cognitive, and physical growth. These milestones assist in identifying potential delays, though it is crucial to remember that each child develops at their own pace [93]. Screening outcomes do not constitute a clear diagnosis but may signal the necessity for a formal developmental assessment by a specialist, who can determine whether the child meets the criteria for ASD [92].

The diagnostic process for ASD encompasses two main components: obtaining a detailed developmental history from parents and observing the child engages with parents and unfamiliar adults during both structured and unstructured evaluations. Ideally, observations of the young child within peer groups contexts like school or daycare would be included in the diagnostic procedure. In a population-based study conducted in the United Kingdom, girls with comparable levels of symptom manifestation to boys were less inclined to acquire a diagnosis of ASD from healthcare services [94]. This observation could indicate that sociocultural factors influence how diagnostic criteria are applied, that girls may possess greater resilience or protective factors that lessen the need for clinical services at similar levels of symptoms, or that there is a need to revise diagnostic tools to better capture traits specific to female autism [95].

The potential for early assessment to identify children with ASD at a young age has piqued researchers’ interest, and several studies have examined the effectiveness of parent-report instruments between the ages of four-teen and twenty-four months, for example the Modified Checklist for Autism in Toddlers (M-CHAT) and the Early Screening of Autistic Traits (ESAT). Nonetheless, there are differing opinions on the level of evidence supporting universal testing across the population, often common as screening [96,97]. It is worth noting that data on the efficacy of treatment interventions in persons diagnosed with ASD by worldwide screening are limited. Furthermore, while it is possible to recognize certain children with ASD before parents or professionals become concerned, diagnosis is missed in many children [98], and most tested groups have not been systematically monitored to detect later-onset autism in children who initially tested negative [99]. Screening often detects children with ASD as well as those with broader developmental concerns developmental impairments. Overall, these measures may be more successful in detecting probable autistic symptoms in high-risk groups, such as young children with older relatives on the spectrum or those sent to community pediatric care for speech or other developmental issues [100].

Furthermore, broad testing might help to raise awareness and early identification of autism symptoms among professionals and the general population. When combined with ongoing developmental monitoring provided by community services, this strategy has the potential to minimize the age at which autism is recognized and diagnosed. These strategies are also relevant for low- and middle-income countries, where ASD and other neurodevelopmental disability testing is still in the early stages of implementation [101]. Limited studies have been conducted on how cultural and ethnic factors influence early autism presentation and parents’ understanding or experience of the condition, which could impact the effectiveness of screening tools and consequently affect both families and children with ASD.

A previous review detailed several widely used screening instruments for ASD [92]. Among them are the Modified Checklist for Autism in Toddlers, Revised with Follow-Up (M-CHAT-R/F), a 20-item questionnaire for children aged six-teen to thirty months, noted for its strong sensitivity and specificity. The Ages and Stages Questionnaire (ASQ) evaluates developmental challenges at specific ages. The Screening Tool for Autism in Toddlers (STAT) is designed for community service providers and comprises 12 items, typically taking around 20 min to complete and focuses on social and communicative behaviors through activities such as imitation and play. The Social Communication Questionnaire (SCQ) includes 40 yes-or-no questions for caregivers to screen for autism in children aged 4 to 40. Additionally, the Parents’ Evaluation of Developmental Status (PEDS) is a parent interview that assesses overall development across various domains to identify potential delays. If screening results raise concerns, pediatricians will refer families to specialists for thorough evaluations, as only trained professionals can provide an official diagnosis.

While significant progress has been achieved in understanding early brain development differences, medical conditions, and behavioral traits linked to later autism diagnoses, the next challenge is translating these findings into validated clinical screening tools. Most current research has focused on potential biomarkers and behavioral precursors that differentiate groups of infants but with less focus on the individual-level predictions of ASD.

The majority of research has targeted infant siblings or those already flagged for concerns, which may not represent the general infant population for broader screening use. Direct comparisons between different biomarker types (e.g., MRI vs. EEG) are challenging, as most studies are conducted in solitude, with few examining combined markers at similar ages [32,35,102]. Often, comparison groups are low-risk infants, potentially inflating prediction accuracy. Additionally, many studies have small, homogeneous samples, limiting generalizability and hindering reliable estimates related to sex, race, and ethnicity. Few studies have explored the impact of comorbid conditions like ADHD on the accuracy of predictions [50,82]. Most of the data have been collected in university laboratories, predominantly from high-income, and racially, and ethnically homogeneous families, rather than in typical community settings where screenings would occur [42,48,72].

## 6. Treatment Methods and Approaches for ASD

### 6.1. Behavioral Interventions

Early social communication skills including eye contact, joint attention, and social referencing are important for early developmental progress and the acquisition of complex abilities [103,104]. These abilities usually arise from ordinary interactions during the first 24 months and are essential for subsequent social, intellectual, and adaptive success [105,106]. Although additional research is required to establish the most effective skills and interventions, Applied Behavior Analysis (ABA) has been beneficial in teaching and retaining these skills [105]. Joint attention, which involves two people sharing their focus on an object, is critical for language development and typically appears between 9 and 15 months [107]. Children with ASD frequently exhibit delays in joint attention, making it a key focus in early intervention programs [108]. Joint attention is divided into two types: responding to and training joint attention, both of which begin with an engaging item or activity but differ in who directs the attention [109]. Initiating joint attention, which is often impaired in children with ASD, entails directing the adult’s focus to share an experience [110]. Interventions that provide stimulating settings and emphasize social reinforcement have been demonstrated to improve joint attention in children with autism [111]. Addressing joint attention with ABA can assist in fostering interactive conversation and shared social experiences.

ABA-based interventions provide a unique approach to addressing language problems in children with ASD by focusing on functional language usage rather than particular words or patterns [112]. Unlike linguistic approaches that assume that learning a word means that it will be used correctly in all contexts, ABA focuses on teaching language based on its function and the circumstances under which it is used. This includes focusing on the verbal operants identified by Skinner: echoics, mands, tacts, and intraverbals [113]. The echoic involves repeating words and is reinforced by social praise or other rewards, laying the groundwork for teaching other verbal skills. Mands are requests for items or information that arise under motivating situations, such as hunger or curiosity, and are essential for communication because they meet immediate needs. In early intervention, mands are often taught initially, with basic requests progressively growing in complexity. The strategy comprises labeling or naming things, activities, or other stimuli, which is reinforced by social interaction [113]. It encourages children to describe their environment and talk about their experiences with others. Finally, the intraverbal is a response to verbal stimuli that do not correspond to the previous input, such as answering questions or filling in missing words [114]. Intraverbals are essential for conversation and social interactions, and they usually emerge after echoic, mand, and tact skills have been established. Additional research is necessary to improve intraverbal training and gain a clearer understanding of the optimal methods for teaching this complicated ability to children with ASD.

Alongside language skills, children with autism frequently encounter challenges with adaptive skills, including daily hygiene and toilet training, which can hinder their independence and increase their vulnerability to abuse [106]. These deficiencies may also limit their social and educational chances. Early intervention programs usually use organized ways to teach adaptive and independent living skills. A task analysis, which defines a thorough step-by-step sequence of actions necessary to execute a task, is an essential intervention approach. This analysis contributes to the development of targeted interventions. These skills are taught using a variety of training procedures: (1) Forward Chaining: This method entails teaching the first step of a task before gradually adding subsequent steps. For example, when teaching handwashing, the child first learns to move the stool, then steps onto it, and so on, with each step added in sequence [115]. (2) Backward Chaining: Start by teaching the task’s last phase, then add the stages that occurred before it in a sequential order. For example, the child starts by drying his or her hands and then moves to previous processes until the full procedure is learned [115]. (3) Total-Task Presentation: Requires the kid to practice all stages of a task every time, enabling them to attempt each step alone before getting reminders or support. Although this approach is thorough, it may take longer than chaining methods [115]. (4) Activity schedules are another useful tool because they include visual aids like images, phrases, or videos to help children through each stage of an activity. These routines can be carried out physically or with technology, such as cellphones or tablets, to give portable and interactive cues [116]. Although practical constraints (such as durability) may restrict exercise plans, technology might enhance their efficacy by offering flexible and engaging cues [117].

### 6.2. Educational Intervention

Music or music therapy has long been used to meet non-musical goals, such as improving social skills, in children with autism [118]. Currently, the bulk of music therapy therapies for ASD are geared toward children and adolescents. These therapies are thought to improve various aspects of social functioning, such as engagement behaviors [119], emotional involvement [120], social interactions [121], social greeting routines [122], joint attention (JA) behaviors [123], peer interactions [124], communication abilities [125], and cognitive social skills [126]. Furthermore, the efficacy of various music therapy treatments for people with ASD might differ. Improvisational music therapy (IMT) is among the most extensively researched music interventions for children with autism [127]. Family-centered music therapy, an important form of IMT, enhances social connections in families, communities, and parent-child relationships [128]. However, studies have produced inconsistent findings or found no change in some areas [129]. Nonetheless, preliminary evidence supports the feasibility of music therapy therapies for children with ASD, at least in terms of enhancing social engagement, verbal communication, behavior initiating, and social-emotional reciprocity. Similarly, engaging children with ASD in play activities might help them form relationships with others and improve their social communication abilities. Patient-centered play therapy is regarded as an effective, evidence-based intervention that tackles fundamental ASD difficulties such as social skills, communication, emotion management, and joint attention, while also reducing repetitive behaviour [130,131].

According to previous research, incorporating parents in interventions boosts their efficacy and skill transfer outside of the school setting [132]. Family-school partnerships (FSPs) take a child-centered approach, working with families and schools to improve results in the social, emotional, behavioral, and academic domains [133]. Active parental participation in education and intervention may have a major influence on children’s learning and development, such as improvements in cognitive and linguistic abilities, school attendance, academic success, and problem-solving ability [134]. Furthermore, parental involvement has been linked to improved prosocial conduct, peer interactions, and self-regulation capacities [135]. Aside from FSPs, which stress collaboration between families and schools, parental involvement (PI) refers to family-level interventions and education for children with ASD. Unlike FSPs, PIs focus on activity planning and execution [136]. Research has shown that adopting the PI model in therapies can improve social communication and reduce limited and repetitive behaviors in children with autism [137].

### 6.3. The Interventions Based on Technological Devices for ASD

With the swift advancement of contemporary technology, various assistive devices for the rehabilitation of individuals with autism have been created and implemented. These devices have demonstrated some effectiveness in ASD interventions and warrant further research and assessment. One such device is the speech-generating device (SGD) is a compact electronic gadget that shows various graphic symbols or written text and produces digital or synthetic speech [138]. For children with autism, who often face challenges with communication, the SGD’s ease of use, the popularity of its output language, and its extensive storage capacity make it a preferred tool [139]. Additionally, the SGD’s functions for making requests, labeling, commenting, and responding to questions enhance its versatility [140]. Research has demonstrated that SGDs can improve communication skills in participants, which is a primary focus in early ASD intervention programs [141].

Similarly, virtual reality (VR) is an advanced and engaging three-dimensional virtual environment created through interactive software and hardware, resulting from a blend of multiple disciplines. As VR technology evolved, researchers were able to effectively treat people with autism [142]. Building on this, immersive virtual reality has been created to better simulate actual objects and settings [143]. Despite these advances, VR still has limits, such as existing VR apps for ASD therapy being very homogeneous and often targeting only one specific feature, with simulated environments that do not entirely replicate reality. It is expected that VR technology will continue to advance, eliminating flaws and addressing the specific needs of children with ASD. In contrast to VR, another more concrete technical innovation—humanoid robots—is also being used in the treatment of autism. Increasing research shows that robotic assistance improves the condition of people with ASD [144]. Unlike humans, robots work inside predictable and well-defined systems, resulting in a highly regulated learning environment that allows for standardized interventions. This organized environment allows people with ASD to focus on important stimuli, and some social behaviors can be reproduced within these controlled interactions [145]. Robots have been employed in autism for a several of reasons, including diagnostics, enhancing eye contact and spontaneous engagement, turn-taking, promoting imitation, emotion identification, shared attention, and encouraging triadic interactions [146]. The encouraging findings of induction training with android robots suggest that investigating alternative robotic intervention techniques may be useful [147].

### 6.4. Medical Treatment and Potential Pharmacological Targets for ASD

Currently, no medications are available worldwide that specifically target the core symptoms of ASD. Instead, existing antipsychotics are commonly used to address associated issues such as anxiety [148], depression [149,150], or obsessive-compulsive disorder [151], which can help alleviate certain ASD symptoms, like ADHD [152,153]. The US Food and Drug Administration (FDA) has authorized risperidone and aripiprazole for the treatment of ASD-related irritability and aggressiveness [154]. Although these drugs can assist with particular ASD-related symptoms, they are not without risks. Aripiprazole, for example, can produce sleepiness, prolonged sleep, and weight gain [155]. Furthermore, selective serotonin reuptake inhibitors (SSRIs), which are already licensed for a variety of diseases, are increasingly being utilized to treat ASD [156]. According to recent reviews of RCTs, medications such as aripiprazole, atomoxetine, bumetanide, and risperidone improve at least one core symptom in children and adolescents, whereas fluoxetine, fluvoxamine, oxytocin, and risperidone benefit adults [156,157]. As a result, discovering common pathways for screening targeted medications remains critical [158,159]. Current clinical studies, for example, are investigating the GABAergic system as a potential therapy strategy for autism [158], while recent research has shown that a specific ERK pathway inhibitor can cure fundamental abnormalities in a mouse model of autism [159].

## 7. The Impact of Early Intervention on ASD Development

Dawson et al. demonstrated the effectiveness of early behavioral therapy in a RCT aimed at alleviating behavioral symptoms in children. The study focused on children aged eight-teen to thirty months who underwent the Early Start Denver Model (ESDM) approach, revealing significant improvements in intelligence quotient (IQ), adaptive behavior and ASD diagnosis in comparison to those receiving community-based interventions. After two years, the ESDM group exhibited an average increase of 17.6 standard score points, whereas the comparison group saw only a 7.0-point improvement from their baseline scores. The adaptive behavior growth rate in the ESDM group aligned more closely with that of typically developing children, while the comparison group experienced more pronounced delays in adaptive behavior. Furthermore, children in the ESDM group were more inclined to transition their diagnosis from ASD to pervasive developmental disorder, not otherwise specified, in contrast to the comparison group [160].

A key concept in autism development is the notion of “sensitive periods” in brain maturation. These critical periods are specific times when the brain is particularly open to learning and skill acquisition, leading to more enduring impacts from repeated exposure to certain stimuli. This heightened receptivity is attributed to the brain’s capacity for stimulation during early developmental stages. Penhune VB noted that during these critical periods, children’s learning is more influenced by frequently encountered stimuli than by those encountered less often [161].

Neuroplasticity describes the brain’s capacity to adapt and create new neural connections throughout an individual’s life, with this capacity being especially pronounced in early childhood (ages two to three) [11]. Early intervention for autism exposes children to a range of learning experiences that can enhance and strengthen these neural connections. This neuroplasticity enables the brain to adjust and reorganize in reaction to therapeutic interventions. By continuously stimulating the brain through targeted interventions, early intervention can effectively influence neural pathways and enhance cognitive and behavioral functioning in children with ASD. Therefore, early intervention that promotes neuroplasticity can help avoid or alter the course of ASD manifestations [162]. Fuller EA et al. reported significant improvements in children’s social communication when early intervention from clinicians indicated that the prognosis for autism could primarily be improved at the initial stages of communication development [163].

## 8. Limitations and Future Directions

This review emphasizes important advances in identifying early indicators and treatment strategies for children with ASD. However, several limitations must be recognized. The variety in research designs, outcomes, and methodology utilized throughout the collected literature makes it difficult to draw broad generalizations regarding the efficacy of therapies. Furthermore, the review does not adequately address potential biases in participant selection or the influence of socio-cultural variables on ASD diagnosis and treatment, which may restrict the findings’ application to various communities.

Future research should establish inclusive and standardized diagnostic criteria that reflect diverse cultural perspectives. Investigating the neurological underpinnings of ASD and the interplay between genetic and environmental factors is essential for targeted interventions. Additionally, integrating technology into therapeutic approaches could enhance engagement and communication skills in children with ASD. Collaboration among researchers, clinicians, and families will be vital in refining intervention strategies and addressing treatment disparities to improve outcomes for children on the autism spectrum.

## 9. Discussion and Concluding Remarks

The increasing prevalence of ASD emphasizes the critical need for early detection and effective intervention strategies. As this review shows, advances in research have provided valuable understanding of the early indicators of ASD, ranging from neurobiological markers detected using MRI and electrophysiological studies to behavioral signs identified in infants. These findings demonstrate that atypical brain development and altered social behaviors frequently occur before a formal diagnosis can be made.

Early identification is critical, as research shows that timely interventions can significantly improve developmental outcomes for children with ASD. ABA programs, as well as innovative approaches such as music therapy and technology-assisted devices, have demonstrated promise regarding the enhancement of social communication and adaptive skills. However, disparities in diagnostic practices and variability in intervention effectiveness necessitate more standardized procedures and personalized treatment plans that consider each child’s unique profile.

Additionally, there is an increasing amount of research supporting the role of environmental factors and genetic underpinnings in ASD encourages further research into prevention strategies and targeted therapies. As we move forward, fostering collaboration among researchers, clinicians, and families will be crucial in improving our insight of ASD and developing comprehensive support systems that empower people on the spectrum. In conclusion, while the complexity of ASD provides considerable obstacles, continued research and a dedication to early intervention provide promise for enhanced outcomes and quality of life for afflicted children and families.

## Data Availability

The raw data supporting the conclusions of this article will be made available by the authors on request.
